# A case of severe headache attributed to vertebral artery dissection

**DOI:** 10.1186/s40981-019-0247-9

**Published:** 2019-04-08

**Authors:** Satoshi Sakakibara, Toshihiko Nakatani, Hanako Yamamoto, Akihiro Motooka, Tatsuya Hashimoto, Yoji Saito

**Affiliations:** 1 Department of Anesthesiology, Shimane University Hospital, 89-1 Enya-cho, Izumo, Shimane Japan; 2 Department of Palliative Care, Shimane University Faculty of Medicine, 89-1 Enya-cho, Izumo, Shimane Japan; 3 Palliative Care Center, Shimane University Hospital, 89-1 Enya-cho, Izumo, Shimane Japan; 4 Department of Anesthesiology, Shimane University Faculty of Medicine, 89-1 Enya-cho, Izumo, Shimane Japan

**Keywords:** Vertebral artery dissection, Severe headache, Magnetic resonance angiography

## Abstract

**Background:**

Vertebral artery dissection (VAD) sometimes has no specific symptoms and is difficult to differentiate from other forms of headache.

**Case presentation:**

A woman in her thirties had a severe, throbbing left-sided headache. A migraine without aura was suspected and zolmitriptan was administered, which alleviated the symptoms. The woman was consequently deemed to have a migraine without aura. Despite the lack of abnormal neurological findings and showed no abnormalities on cranial computed tomography, her symptoms were not typical for migraines and showed little improvement with therapy. She therefore underwent a cranial magnetic resonance imaging (MRI) examination, which revealed VAD, for which she was transferred to the department of neurosurgery for conservative treatment.

**Conclusion:**

The possibility of vertebral artery dissection should be considered in the differential diagnosis of severe secondary headaches, and prompt diagnosis and treatment based on detailed MRI and magnetic resonance angiography examinations should be performed.

## Background

Vertebral artery dissection (VAD) often occurs in young people and can lead to subarachnoid hemorrhage and/or cerebral infarction, both of which can be life-threatening. Even if the patient’s life can be saved, they might develop serious sequelae. For this reason, VAD requires early diagnosis and treatment [1]. In recent years, advances in diagnostic imaging have led to an increase in the number of case reports of asymptomatic VAD and VAD with just minor subjective symptoms, such as headache and dizziness [2]. While sudden headache, neck pain, and facial pain are common initial symptoms, VAD has no specific symptoms and is difficult to differentiate from other forms of headache, such as migraine [3]. This report describes the case of a patient we encountered, whose severe headache was caused by VAD.

## Case presentation

The patient was a female in her thirties who visited her local clinic complaining chiefly of pain in the left occipital to temporal regions of the head, rotational vertigo, and vomiting. Although she had a history of depression and floating dizziness, her symptoms had subsided in recent years and were not a hindrance to her daily life. There was no family history of inheritance vascular wall disease.

On the morning of the day of symptom onset, she developed rotational vertigo of no apparent cause. In the afternoon, she developed pain in the left occipital to temporal regions of the head while driving and subsequently started vomiting in the night. Hence, she visited her local neurology clinic on the second day of symptom onset and underwent a computed tomography (CT) scan of the head and plain radiography of the neck; however, there were no obvious abnormal findings and a diagnosis of suspected herpes zoster was made. The woman was prescribed with valaciclovir, pregabalin, and acetaminophen but with no symptom improvement. Carbamazepine and aspirin that were added to the prescription were also ineffective and, hence, she was referred to our anesthesiology pain clinic for examination.

The patient had a severe headache during the examination, which she rated 100 mm on a visual analog scale (VAS). Every few hours, she developed throbbing, pulsatile pain that prevented her from sleeping and rendered her practically bedridden during the day. The associated vomiting also prevented her from eating. There was no allodynia of the head, trigeminal paresthesia, or motor symptoms in the region of the facial nerve. She also showed no other abnormal neurological findings in the spinal nerve region and had no skin rash over the left occipital to temporal regions of the head. There were no bulbar conjunctival congestion, fever, or symptoms of meningeal irritation. Her blood pressure was, however, elevated to 160/100 mmHg.

Her symptoms were considered to be in line with the diagnostic criteria for migraine without aura according to the International Classification of Headache Disorders 3rd edition (Beta version) [4], defined as severe unilateral, pulsatile headaches that come in cycles every few hours. The headaches were also accompanied by vomiting. Hence, she was prescribed with 2.5 mg of zolmitriptan. At this point, although the therapeutic effect was inadequate, another zolmitriptan dose of the same amount led to an improvement of her symptoms. Treatment was consequently commenced for migraine without aura. However, since the severe headache was sudden and unprecedented, a differential diagnosis of secondary headache was also simultaneously made.

**Fig. 1:**
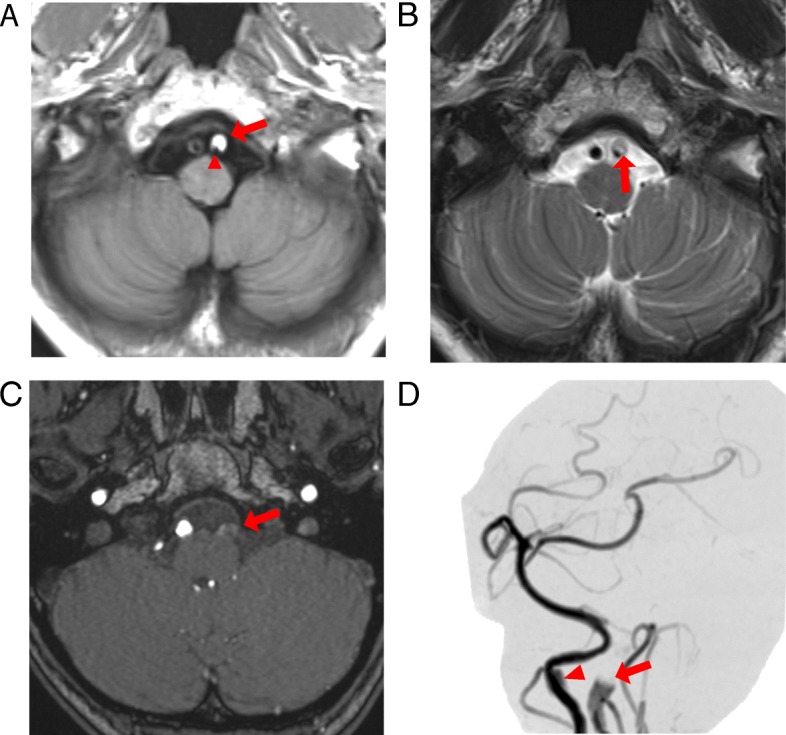
3T magnetic resonance imaging of the head. **a** T1-weighted images showed a high-signal intensity lesion (arrow) and narrowing of the residual signal void (arrowhead) of the left vertebral artery. **b** T2-weighted images showed a curvilinear high-signal structure and an intimal flap (arrow), suggesting left vertebral artery dissection and intramural hematoma. **c** and **d** Three-dimensional time-of-flight magnetic resonance angiography showed obstruction of the left vertebral artery (arrow), an aneurysm related to dissection of the right vertebral artery (arrowhead)

Treatment after hospitalization involved regular oral lomerizine with zolmitriptan and loxoprofen as needed. From day 2 after admission, the patient showed a decrease in her VAS score to 50 mm. She nonetheless continued to experience recurrent, severe headache attacks rated 100 mm on the VAS every few hours. Despite the partial efficacy of as-needed use of zolmitriptan, loxoprofen had both a better effect and longer duration of action. Finally, the persistent high blood pressure despite the apparent improvement in her symptoms led us to consult the department of cardiology on day 3, after which antihypertensive therapy with calcium antagonists was commenced. Furthermore, although secondary headache caused by intracranial disease had been ruled out because the patient had been referred to us by a neurology clinic and showed no abnormalities on cranial CT, we decided to perform magnetic resonance imaging (MRI) and magnetic resonance angiography (MRA) on day 8 because of the inadequate effect of zolmitriptan on the migraines and the atypically long duration of the headaches. Imaging revealed the findings of left VAD and occlusion, right VAD and an aneurysm related to dissection (Fig. 1). There was absence of ischemic change in the brain on the imaging. Since she required specialist treatment for the VAD, she was transferred to the department of neurosurgery, where treatment with fluid replacement and oral antiplatelet drugs was commenced. The headache in the present case met the diagnostic criteria for headache caused by VAD in the International Classification of Headache Disorders 3rd edition (Beta version) [4]. The headaches subsequently subsided for a short time and her condition stabilized, although she once again presented with an increase in headaches on day 18, together with further vertigo, nausea, vomiting, motor ataxia (positive result in the finger-to-nose test), hiccups, hoarseness, dysphagia, and thermal hypoalgesia in the right upper and lower limbs, suggesting the development of Wallenberg syndrome. Repeat MRI examination revealed progression of the left VAD, occlusion of the left posterior inferior cerebellar artery, and findings suggestive of cerebral infarction in the left cerebellum and lateral medulla oblongata. She was treated conservatively with edaravone, argatroban, and glycerine, and was discharged home with ongoing rehabilitation and was reintegrated into society 6 months after symptom onset.

## Discussion

VAD can develop into a subarachnoid hemorrhage if the dissected section ruptures, and into cerebral infarction if vascular occlusion occurs in the dissected section, making it one of the most common causes of stroke in young people [5]. In Japan, a nationwide survey related to vertebrobasilar artery dissection found that bleeding and ischemia occur in as many as 30.5% and 33.1% of cases, respectively [6]. The vertebral artery branches into the posterior inferior cerebellar artery, anterior inferior cerebellar artery, superior cerebellar artery, and basilar artery, among others, which supply blood to the brainstem, cerebellum, cervical spinal cord, and other regions whose nourishment is directly linked to survival. Stroke caused by VAD can therefore be fatal. However, even if cases such as the present are not fatal, occlusion of the posterior inferior cerebellar artery can result in Wallenberg syndrome and other conditions that may cause serious after-effects. Originally recognized as a relatively rare disease, VAD has since been confirmed in cases with few symptoms, or without any symptoms at all, thanks to recent advances in diagnostic imaging, such as MRI, MRA, and CT angiography (CTA) [2]. Recent imaging techniques have also shown that some patients with VAD are asymptomatic and remain undiagnosed and that this disease is much more common than originally believed.

The causes of VAD are classified as traumatic and non-traumatic; even minor movements such as extending or rotating the neck can cause dissection [1]. In the present case, the headache developed while driving a car, so it is likely that extension and rotation of the neck while driving was the cause of the VAD. The symptoms of VAD are likely related to cerebral infarction, but many cases of headache alone have also been recognized [2]. The headache caused by the VAD likely results from the direct tear in the blood vessel wall. Blood vessels, like arteries, are known to have many nerves surrounding them. Irritation of the wall likely leads to a cascade of events, including pro-inflammatory neurotransmitters from the nerve terminals that surround the blood vessel. This may cause pain away from the actual site of the dissection [7]. In such cases, differentiating VAD from migraine is difficult [8]. Besides headache, our patient also had nausea and rotational vertigo as symptoms, but with no abnormal neurological or CT findings. Furthermore, a migraine was suspected because administration of zolmitriptan improved the headache. However, since the sudden unprecedented headache was suspected to be a secondary headache [9], the decision was made to commence treatment for migraine while simultaneously trying to differentiate the cause of secondary headache. Diagnosis of VAD is difficult with simple CT alone; additional MRI, MRA, or CTA is reportedly useful [10]. Stroke often occurs within several days or weeks of VAD and can be fatal or result in serious long-term sequelae. Diagnosis and treatment should, therefore, commence as soon as possible [11].

## Abbreviations

CT: Computed tomography
CTA: Computed tomography angiography
MRA: Magnetic resonance angiography
MRI: Magnetic resonance imaging
VAD: Vertebral artery dissection
VAS: Visual analog scale
